# Adrenal gland adenoma with myelolipoma in a canine patient: a rare case report and diagnostic insights

**DOI:** 10.3389/fvets.2026.1780830

**Published:** 2026-03-19

**Authors:** Ecaterina Semzenisi, Romelia Pop, Andrada Negoescu, Lucia Bel, Alexandra Creț, Irina Constantin, Claudiu-Nicusor Ionica, Sorana Catoi, Ibrahima Mamadou Sall, Alexandu-Flaviu Tăbăran

**Affiliations:** 1 Department of Pathology, Faculty of Veterinary Medicine, University of Agricultural Sciences and Veterinary Medicine of Cluj-Napoca, Cluj-Napoca, Romania; 2 Department of Anesthesiology and Surgical Propedeutics, University of Agricultural Sciences and Veterinary Medicine of Cluj-Napoca, Cluj-Napoca, Romania; 3 Department of Nutrition, Faculty of Veterinary Medicine, University of Agricultural Sciences and Veterinary Medicine of Cluj-Napoca, Cluj-Napoca, Romania

**Keywords:** adenoma, adrenal gland, histology, immunohistochemistry, myelolipoma

## Abstract

Adrenal gland tumors are uncommon in dogs, and the simultaneous presence of epithelial and mesenchymal neoplasms within a single adrenal gland is exceptionally rare. We describe a rare case of an adrenocortical adenoma associated with a myelolipoma in a 7-year-old male Pekingese dog presented with acute abdominal pain and fever. Diagnostic imaging identified a well-defined mass in the right adrenal gland, with no evidence of vascular invasion or metastatic disease. Surgical adrenalectomy was subsequently performed. Histopathological evaluation revealed a well-circumscribed adrenocortical adenoma containing an intratumoral myelolipomatous component composed of mature adipose tissue and trilineage hematopoietic elements. Immunohistochemical analysis demonstrated Melan A positivity in the adenomatous component, while the myelolipomatous tissue showed diffuse vimentin expression and lacked Melan A immunoreactivity. The postoperative course was uneventful, and no recurrence was detected at one-year follow-up. This case underscores the diagnostic complexity of heterogeneous adrenal masses in dogs and highlights the essential role of histopathology and immunohistochemistry in the accurate identification of rare mixed adrenal lesions.

## Introduction

Adrenal gland tumors in dogs are relatively uncommon but represent a significant clinical challenge due to their diverse presentations and potential for substantial systemic effects (1). Among these, adrenocortical adenomas are benign neoplasms that arise from the adrenal cortex, often characterized by their functional capacity to produce excess hormones such as cortisol, leading to hyperadrenocorticism. However, adrenal adenomas may also be non-functional, remaining clinically silent until they reach a considerable size or are incidentally discovered during imaging for unrelated conditions (2). Conversely, myelolipomas are rare, benign, non-functioning tumors composed of mature adipose tissue and hematopoietic elements (3). While myelolipomas are more commonly reported in humans, they have also been occasionally documented in veterinary medicine, particularly dogs. These tumors are typically asymptomatic and are usually discovered incidentally during imaging or necropsy (4).

The coexistence of an adrenocortical adenoma with a myelolipoma within the same adrenal gland is an exceedingly rare phenomenon in both human and veterinary medicine. The pathogenesis of this combination remains unclear, but it is speculated that chronic adrenal hyperstimulation or metaplastic processes may play a role (5). The clinical implications of this dual pathology are not fully understood; however, the presence of a myelolipoma generally does not alter the behavior of the adrenal adenoma (6). In this report, we present a rare case of a dog diagnosed with an adrenal gland adenoma with coexisting myelolipoma. We discuss the clinical presentation, diagnostic workup, surgical findings, and histopathological features of this rare case, as well as the challenges and considerations in managing such a condition.

## Case description

### Signalment and history

A 7-year-old male Pekingese dog with a previous history of pancreatitis was presented to the University of Veterinary Medicine in Cluj-Napoca, Romania.

### Clinical examination and laboratory findings

The patient presented with non-specific clinical signs, acute abdominal pain, and fever. On clinical examination, the dog showed signs consistent with an acute abdomen and pyrexia. Hematological analysis revealed leukopenia, with a leukocyte count of 1.53 × 10^9^/L (reference range: 6.0–17.0 × 10^9^/L), and neutropenia, with an absolute neutrophil count of 0.04 × 10^9^/L (reference range: 3.0–11.5 × 10^9^/L). These hematological abnormalities were interpreted as consistent with an acute systemic inflammatory response associated with the clinical presentation. Serum biochemistry demonstrated increased alkaline phosphatase activity (ALKP 520 U/L, reference range: 23–212 U/L).

### Diagnostic imaging

**Figure 1:**
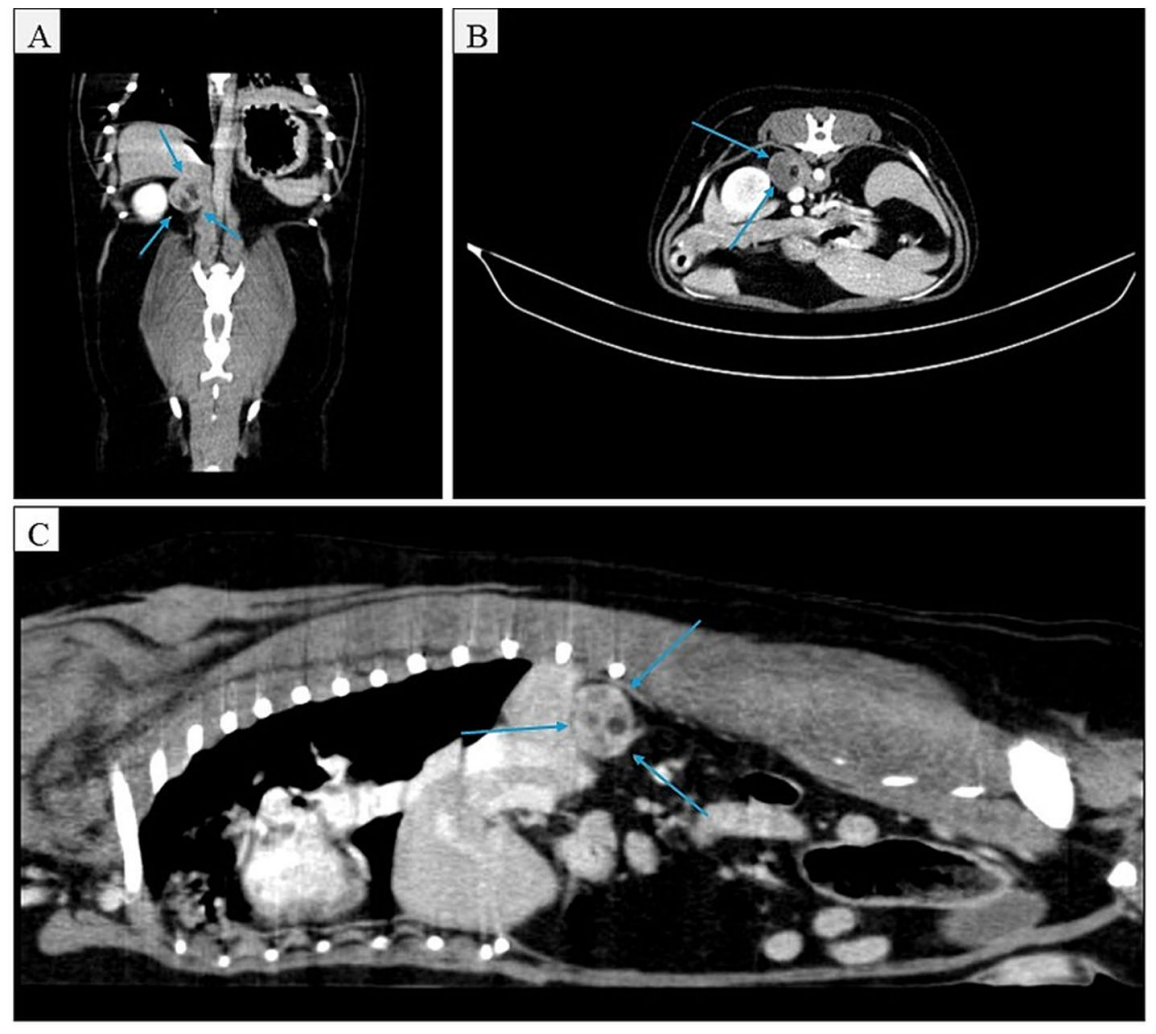
Contrast-enhanced computed tomography (CT) images showing an enlarged right adrenal gland (blue arrows) in different planes: **(A)** coronal reconstruction, **(B)** transverse (axial) image, and **(C)** sagittal reconstruction. The lesion showed mild heterogeneous contrast enhancement and caused displacement, but no invasion, of adjacent vascular structures. No evidence of vascular thrombosis or distant metastasis was observed.

Abdominal ultrasonography revealed a mass located in the right cranial quadrant of the abdomen, and a computed tomography (CT) examination was subsequently recommended. Contrast-enhanced CT identified a 2.1 cm diameter mass occupying the topography of the right adrenal gland. No evidence of regional lymph node involvement or vascular invasion was identified (Figure 1).

### Surgical treatment

Adrenalectomy was performed through a midline incision; due to the right-sided location of the mass, a paracostal approach was also used. Preoperatively, cefuroxime (22 mg/kg, IV) was administered as antimicrobial prophylaxis, and dexamethasone (1 mg/kg, IV) was given to prevent postoperative hypoadrenocorticism.

The animal was anesthetized using midazolam (0.2 mg/kg, IV) and methadone (0.2 mg/kg, IV), with propofol administered to effect for endotracheal intubation. Isoflurane was used for anesthesia maintenance. Dopamine and crystalloids were administered intraoperatively to manage hypotension.

To expose the adrenal gland tumor, the duodenum was mobilized toward the left kidney, allowing visualization of the right liver lobes and right kidney. The neoplastic adrenal gland was removed via gentle dissection. Hemostasis was achieved using a LigaSure^™^ Vessel Sealing System (Medtronic, United States), vascular metallic clips (Hemoclips^®^, Aesculap^®^, B. Braun, Germany), and 4-0 monofilament absorbable suture material (Monocryl^®^, Ethicon, Johnson & Johnson). The tumor had not invaded the kidney, and no thrombus was observed in the renal or phrenic veins or in the caudal vena cava. Only the adrenal gland was submitted for histopathological examination. The abdomen was closed routinely.

### Postoperative course and follow-up

Postoperative analgesia during the first 24 h consisted of buprenorphine (0.02 mg/kg, IV, TID), metamizole (25 mg/kg, IV, BID), robenacoxib (2 mg/kg, SC), and lidocaine administered as a continuous rate infusion (20 μg/min). Metamizole and buprenorphine were continued for 5 days, and robenacoxib was administered for 7 days.

The patient showed progressive clinical improvement and was hospitalized for 5 days following surgery. At a one-year follow-up, the dog was bright and alert, and no mass was detected on ultrasonographic examination.

**Figure 2:**
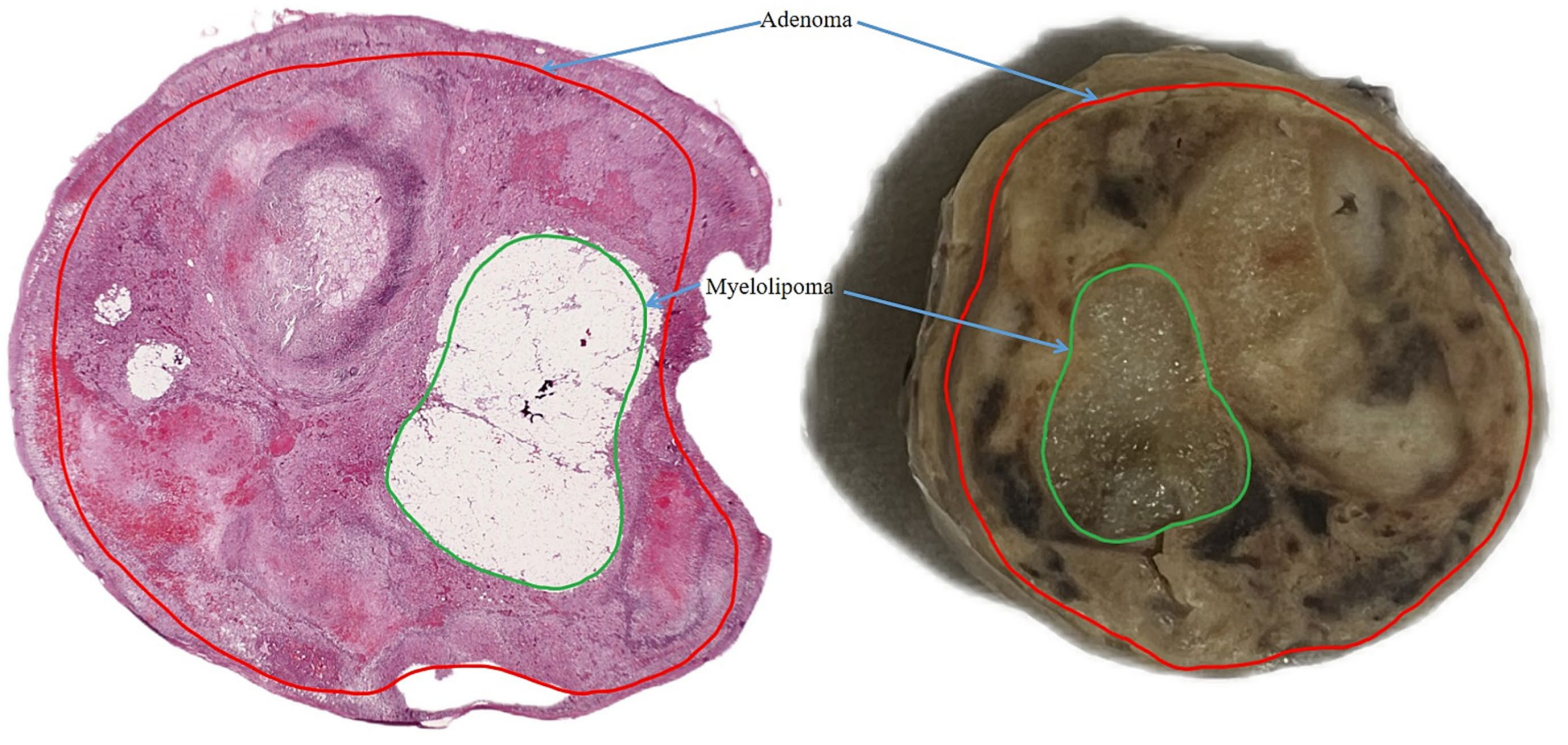
Right adrenal gland dog. On cross-section: the right adrenal gland showed a heterogeneous appearance with areas of necrosis intermixed with hemorrhagic foci, mature adipose tissue, and compact, well-circumscribed nodules consistent with an adrenal adenoma. The lesion exhibited a clear demarcation between the fatty components typical of myelolipoma and the solid, yellow-tan tissue characteristic of the adenoma. No invasion into surrounding structures is noted. Scale bar: 1 cm.

Gross description: On cross-section, the right adrenal gland showed a heterogeneous appearance. The cut surface revealed areas of necrosis intermixed with hemorrhagic foci, extensive mature adipose tissue, and compact, well-circumscribed nodules consistent with an adrenal adenoma. Multiple areas containing adipose tissue were sampled for histopathological evaluation in order to confirm the presence of hematopoietic elements within the fatty component. The lesion exhibited a clear demarcation between the fatty components typical of myelolipoma and the solid, yellow-tan tissue characteristic of the adenoma. No invasion into surrounding structures was noted (Figure 2).

### Histopathological examination

**Figure 3:**
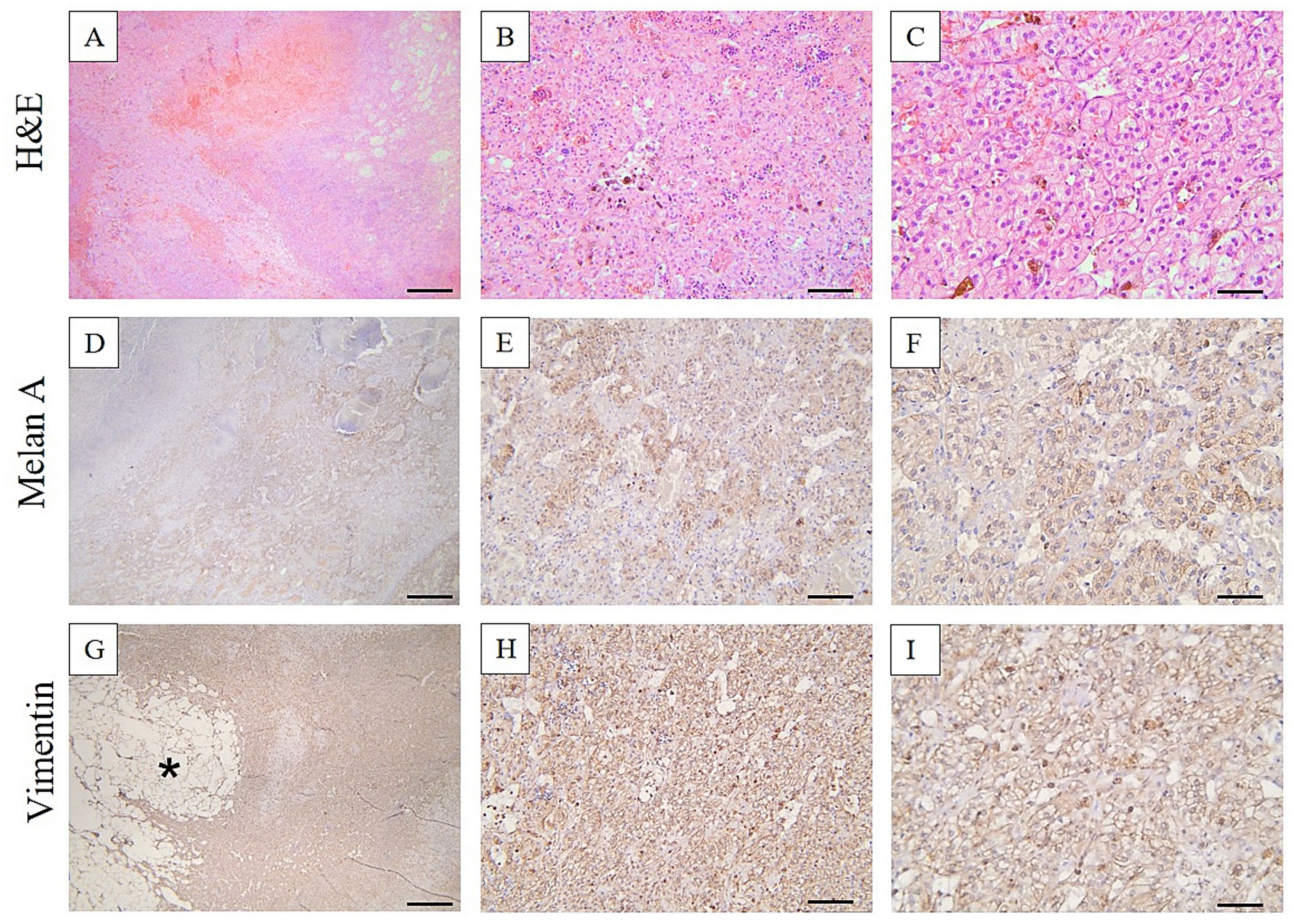
Histologic section of the adrenal gland adenoma. The normal architecture of the gland was replaced by a moderately well-defined, partially encapsulated compressive mass. This mass comprised a peripheral layer of adrenal cortical epithelial cells from the zona fasciculata. These cells are polygonal in shape, containing abundant vacuolated cytoplasm and large, round vesicular nuclei. The neoplastic cells demonstrated positive cytoplasmatic immunoexpression for Melan A and vimentin. **(B,E,H)** Illustrate the neoplastic adrenocortical adenoma component. **(C,F,I)** Show the residual adrenal cortical cells of the zona fasciculata. H&E stain **(A–C)**, Melan A **(D–F)**, vimentin **(G–I)**. Ob. 4× **(A,D,G)**, Ob. 20× **(B,E,H)**, Ob. 40× **(C,F,I)**. Scale bar 50 μm **(C,F,I)**, 100 μm **(B,E,H)**, and 500 μm **(A,D,G)**.

**Figure 4:**
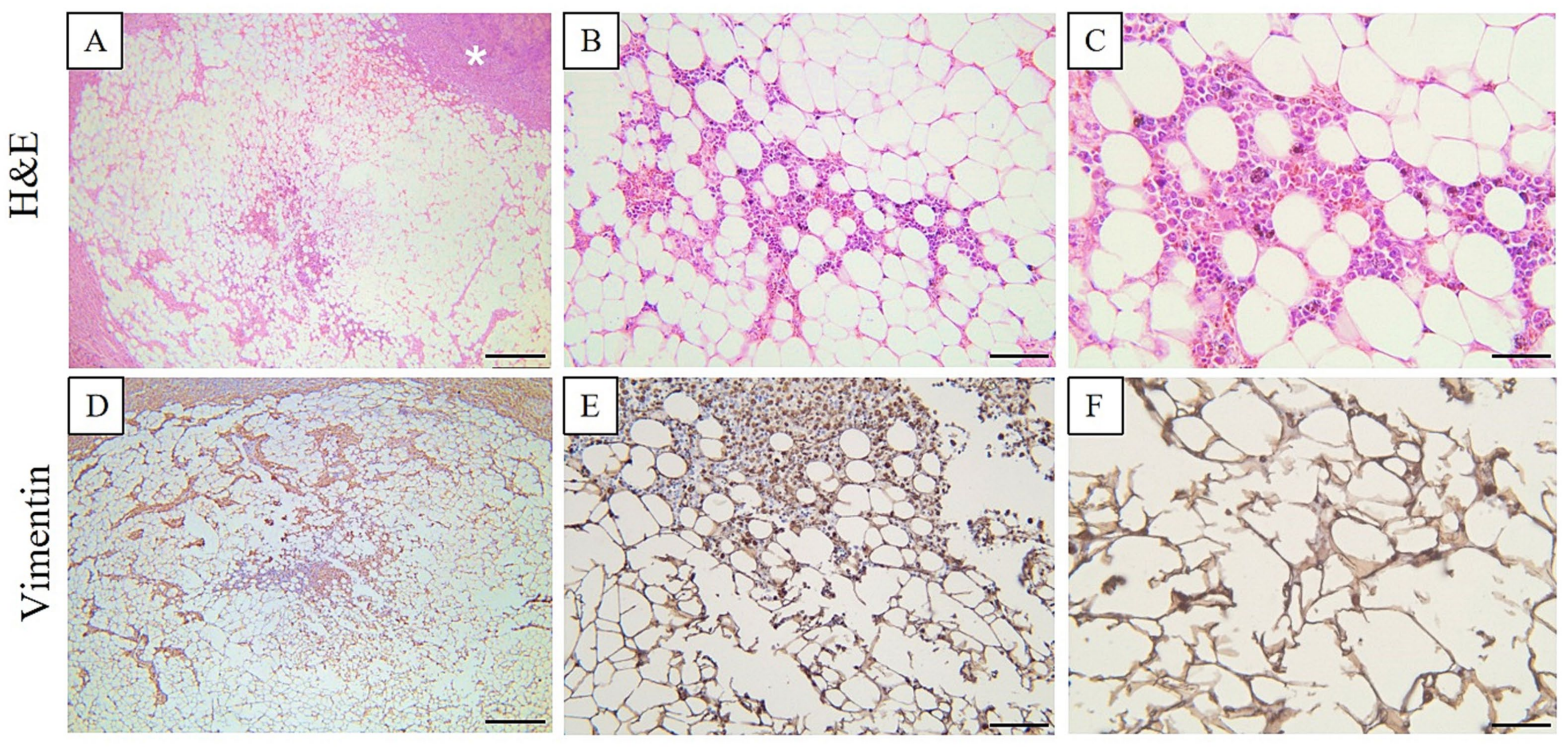
Histologic section of the adrenal gland myelolipoma. The central region of the gland displayed an irregular, non-encapsulated proliferation of well-differentiated adipocytes, interspersed with varying amounts of myeloid precursors, erythroid precursors, and megakaryocytes. The cells showed negative cytoplasmatic immunolabeling for Melan A while demonstrating a positive immunoexpression for vimentin. H&E stain **(A–C)**, and vimentin **(D–F)**. Ob. 4× **(A,D)**, Ob. 20× **(B,E)**, Ob. 40× **(C,F)**. Scale bar 50 μm **(C,F)**, 100 μm **(B,E)**, and 500 μm **(A,D)**. Additional immunohistochemical staining with MPO and CD34 was performed but did not demonstrate specific immunoreactivity.

For histology, the right adrenal gland was fixed in 10% neutral buffered formalin (NBF) and embedded in paraffin following the routine processing protocol. Two-micrometer histological sections were stained using hematoxylin and eosin (H&E). The histological samples were further examined by immunohistochemistry using a Leica Bond-Max automated immunostainer (Leica Microsystems) for the tissular expression for Melan A (mouse monoclonal antibody, clone A103, Leica Biosystems, ready to use) and vimentin (mouse monoclonal antibody, clone V9, Leica Biosystems, ready to use). The immunohistochemistry protocol using a polymer-based detection system (Leica Biosystems, Nr. DS9800) having as a chromogen 3,3-diaminobenzidine (DAB) was carried out by the protocols provided by the auto-immunostainer producer. The histologic section of the right adrenal gland reveals that the normal glandular architecture is disrupted by a moderately well-defined, partially encapsulated mass. This mass compresses the surrounding tissue. At the periphery of the mass, there is a thin layer of adrenal cortical epithelial cells, specifically from the zona fasciculata. These epithelial cells are polygonal in shape, featuring abundant, vacuolated cytoplasm and large, round nuclei with a vesicular appearance. The central region of the mass lacks encapsulation and exhibits a disorganized proliferation of mature adipocytes. Interspersed among these adipocytes are varying numbers of myeloid and erythroid precursors, along with megakaryocytes, indicating a mixed cellular composition within this portion of the mass. Immunohistochemically, the cells of the adrenal gland adenoma exhibited strong diffuse cytoplasmic immunolabeling for Melan A and vimentin. In contrast, in myelolipoma, the cells showed a negative immunoexpression for Melan A, while demonstrating strong and diffuse immunolabeling for vimentin (Figures 3, 4). Additional immunohistochemical staining using antibodies against myeloperoxidase (MPO; rabbit monoclonal antibody, clone SP72, Cell Marque Sigma Aldrich, ready to use) and CD34 (mouse monoclonal antibody, clone QBEnd/10, Leica Biosystems, ready to use) was performed in order to further characterize the hematopoietic component of the lesion. However, both markers failed to demonstrate specific immunoreactivity in the examined tissue sections and the staining was considered non-specific.

## Discussion

Adrenal gland tumors in dogs comprise a diverse group of lesions with a wide spectrum of clinical behavior, ranging from incidental, non-functional findings to hormonally active or locally invasive masses. Among benign adrenal neoplasms, adrenocortical adenomas are relatively well recognized in veterinary practice, whereas adrenal myelolipomas remain distinctly uncommon. The coexistence of an adrenocortical adenoma and a myelolipoma within the same adrenal gland, as observed in the present case, is exceptionally rare and has only sporadically been described in both human and veterinary literature (6–9).

Adrenocortical adenomas originate from the adrenal cortex and may be either functional or non-functional. Functional adenomas are most commonly associated with excessive hormone production, particularly cortisol, resulting in hyperadrenocorticism. In contrast, non-functional adenomas are frequently discovered incidentally during imaging studies performed for unrelated clinical indications. In the present case, the dog showed no clinical or laboratory findings suggestive of hormonal hypersecretion.

Given the acute presentation characterized by abdominal pain, fever, leukopenia, neutropenia, and increased alkaline phosphatase activity, these findings were interpreted in the context of an acute inflammatory process rather than a chronic endocrine disorder. No classical clinical signs of hyperadrenocorticism, such as polyuria, polydipsia, bilateral symmetrical alopecia, or muscle wasting, were observed. Therefore, endocrine function tests, including the low-dose dexamethasone suppression test (LDDST) and urine cortisol-to-creatinine ratio (UCCR), were not performed. Thyroid palpation was conducted as part of the routine clinical examination and revealed no abnormalities. Dedicated thyroid imaging or hormonal testing was not pursued due to the absence of clinical signs suggestive of thyroid dysfunction. This represents a limitation of the present case. These findings support the diagnosis of a non-functional adrenocortical adenoma, consistent with previous reports of clinically silent adrenal adenomas detected incidentally (10).

Myelolipomas are benign mesenchymal tumors characterized by a mixture of mature adipose tissue and hematopoietic elements, including myeloid and erythroid precursors as well as megakaryocytes. In veterinary medicine, myelolipomas are most commonly reported as incidental findings at necropsy or during diagnostic imaging and are typically asymptomatic and non-functional. The pathogenesis of adrenal myelolipomas remains incompletely understood. Proposed mechanisms include metaplastic transformation of adrenal cortical stromal cells, chronic adrenal stimulation, or differentiation of resident mesenchymal stem cells. In human medicine, myelolipomas have been associated with endocrine disorders, chronic stress, and prolonged hormonal stimulation, factors that may also contribute to their development in animals (11, 12). In order to further characterize the hematopoietic component of the lesion, additional immunohistochemical staining using MPO and CD34 was performed. However, these markers did not demonstrate specific immunoreactivity in the examined tissue sections. This lack of staining may be related to limited cross-reactivity of certain antibodies in canine tissues, which has been reported as a limitation in veterinary immunohistochemistry.

The concurrent presence of an adrenocortical adenoma and a myelolipoma within the same adrenal gland raises important questions regarding their potential pathogenetic relationship.

Several reports in the veterinary literature have described adrenal myelolipomas either as isolated lesions or in association with other adrenal abnormalities. For example, adrenal myelolipoma has been reported as an incidental finding during pathological examination in a dog (7), while other studies have described adrenal adenomas associated with myelolipomatous changes detected during diagnostic imaging (6). In most of these cases, myelolipomas were considered incidental lesions and were not associated with clinically significant endocrine dysfunction. Similarly, the dog in the present report did not show typical clinical signs of hyperadrenocorticism, and the adrenal mass was detected during the diagnostic investigation of acute abdominal signs rather than endocrine disease.

In contrast to previously reported cases, where myelolipomatous tissue was described as a separate lesion or associated with multiple adrenal abnormalities, the lesion described in the present case consisted of a well-defined adrenocortical adenoma containing a myelolipomatous component within the same adrenal gland. Histologically, the epithelial adenomatous component and the adipose–hematopoietic component were clearly demarcated and showed different immunohistochemical profiles, supporting the interpretation of two histogenetically distinct tissue components.

The coexistence of two different tumor components within the same anatomical location may raise the question of a possible collision tumor. However, the present case does not strictly meet the classical definition of this entity. Collision tumors are typically defined as the coexistence of two independent malignant neoplasms within the same anatomical site without histological intermixing (13). In contrast, both lesions identified in this case were benign and consisted of an epithelial adrenocortical adenoma and a mesenchymal myelolipoma. Nevertheless, the rare simultaneous occurrence of these two tumor types raises similar questions regarding their possible pathogenetic relationship. Mechanisms such as chronic adrenal stimulation, local microenvironmental changes, or stromal metaplasia have been proposed as potential explanations for the development of myelolipomatous lesions within the adrenal gland.

One hypothesis suggests that chronic stimulation or local microenvironmental alterations induced by the adenoma may promote metaplastic changes in stromal elements, ultimately leading to myelolipoma formation. Alternatively, the two lesions may represent independent and coincidental processes arising within the same gland. In the present case, the sharp histological separation between the epithelial adenomatous component and the adipose–hematopoietic myelolipomatous component, together with their distinct immunohistochemical profiles, supports the interpretation of two coexisting but histogenetically distinct lesions (12).

From a diagnostic standpoint, adrenal masses containing both soft tissue and fat components present a particular challenge. Differential diagnoses for fat-containing adrenal lesions include lipoma, liposarcoma, extramedullary hematopoiesis, and pheochromocytoma with fatty degeneration. In this case, contrast-enhanced computed tomography revealed a well-defined adrenal mass without evidence of vascular invasion or distant metastasis, findings that favored a benign process. Although the identification of fat-attenuating areas on CT may raise suspicion for a myelolipoma, definitive diagnosis relies on histopathological examination. In the present case, the combination of routine histology and immunohistochemistry was crucial for accurate tumor characterization, with Melan A immunoreactivity confirming the adrenocortical origin of the adenoma and the lack of Melan A expression in the myelolipomatous component supporting its mesenchymal nature.

Surgical excision remains the treatment of choice for adrenal masses when clinical signs are present, when malignancy cannot be excluded, or when there is a risk of local complications. In the present case, adrenalectomy was indicated due to the acute clinical presentation and the presence of an adrenal mass with uncertain biological behavior. The absence of vascular invasion or thrombus formation allowed complete surgical excision, and the postoperative course was uneventful. Long-term follow-up at 1 year revealed no evidence of recurrence, further supporting the benign nature of both lesions.

Clinically, this case emphasizes the importance of considering complex and mixed adrenal lesions in the differential diagnosis of adrenal masses in dogs. It also highlights the value of a multimodal diagnostic approach that integrates imaging, surgical findings, histopathology, and immunohistochemistry to achieve an accurate diagnosis. Although myelolipomas are generally regarded as incidental findings, their coexistence with other adrenal neoplasms may complicate diagnostic interpretation and influence clinical decision-making.

A limitation of the present study is that immunohistochemical confirmation of megakaryocytic differentiation using CD61 was not performed because this antibody was not available in our laboratory at the time of analysis. Nevertheless, megakaryocytes were readily identifiable based on their characteristic morphology on routine hematoxylin and eosin–stained sections, together with myeloid and erythroid precursors, supporting the diagnosis of myelolipoma.

In conclusion, this report describes a rare case of concurrent adrenocortical adenoma and myelolipoma within the same adrenal gland in a dog. This case adds to the limited veterinary literature on mixed adrenal lesions and underscores that benign epithelial and mesenchymal tumors may coexist within the adrenal gland. Awareness of this possibility is important for both clinicians and pathologists, particularly when evaluating heterogeneous adrenal masses with adipose components on imaging.

## Data Availability

The original contributions presented in the study are included in the article/supplementary material, further inquiries can be directed to the corresponding author.
