# Correction: Adrenal gland adenoma with myelolipoma in a canine patient: a rare case report and diagnostic insights

**DOI:** 10.3389/fvets.2026.1853921

**Published:** 2026-04-30

**Authors:** Ecaterina Semzenisi, Romelia Pop, Andrada Negoescu, Lucia Bel, Alexandra Creţ, Irina Constantin, Claudiu-Nicusor Ionica, Sorana Catoi, Ibrahima Mamadou Sall, Alexandu-Flaviu Tǎbǎran

**Affiliations:** 1 Department of Pathology, Faculty of Veterinary Medicine, University of Agricultural Sciences and Veterinary Medicine of Cluj-Napoca, Cluj-Napoca, Romania; 2 Department of Anesthesiology and Surgical Propedeutics, University of Agricultural Sciences and Veterinary Medicine of Cluj-Napoca, Cluj-Napoca, Romania; 3 Department of Nutrition, Faculty of Veterinary Medicine, University of Agricultural Sciences and Veterinary Medicine of Cluj-Napoca, Cluj-Napoca, Romania

**Keywords:** adenoma, adrenal gland, histology, immunohistochemistry, myelolipoma

In the published article, there was an error in the **Funding** statement. The **Funding** statement was incorrectly given as:

“The author(s) declared that financial support was received for this work and/or its publication. This study was supported by the University of Agricultural Sciences and Veterinary Medicine of Cluj-Napoca.”

The correct **Funding** statement is:

“The author(s) declared that financial support was received for this work and/or its publication. This publication was supported by Metup SRL within the ZoeSoma project. The funder was not involved in the study design, collection, analysis, interpretation of data, the writing of this article, or the decision to submit it for publication.”

The original version of this article has been updated.

